# Clinical Risk and Medico-Legal Implications in Zygomatic Implant Rehabilitation: An Umbrella Review of Systematic Reviews

**DOI:** 10.3390/diagnostics16060901

**Published:** 2026-03-18

**Authors:** Francesco D’Ambrosio, Alfonso Acerra, Elena de Laurentiis, Antonio Babino, Alessandro Santurro, Francesco Giordano

**Affiliations:** 1 Department of Medicine, Surgery and Dentistry, University of Salerno, 84084 Salerno, Italy; e.delaurentiis@studenti.unisa.it (E.d.L.); asanturro@unisa.it (A.S.); frgiordano@unisa.it (F.G.); 2 Private Practitioner, 84035 Polla, Italy; antonio.babino@gmail.com

**Keywords:** dental implant, zygomatic implants, zygomatic complications

## Abstract

**Background**: Zygomatic implants (ZIs) were initially pioneered by Brånemark to rehabilitate patients suffering from destructive diseases through original surgical technique (OST). Subsequently, other techniques were proposed, such as the zygomatic anatomy-guided approach (ZAGA). This umbrella review was conceived to quantify and critically characterize the spectrum of complications associated with different techniques of ZI placement. **Methods**: Systematic reviews, encompassing both those with and without meta-analysis, focusing on the complications associate with ZIs and published only in the English language were systematically sought. A systematic literature search was performed through MEDLINE/Pubmed, Scopus, BioMed Central, and the Cochrane Library, and the PROSPERO register. **Results**: A total of 11 articles were included. The latter documented the spectrum of complications associated with ZIs, ranging from minor morbidities such as sinusitis, hematoma, and soft tissue complications up to severe adverse events such as orbital penetration and diplopia. **Conclusions**: The use of described ZI OST and ZAGA in cases of severe maxillary resorption is associated with a high implant survival rate and a low incidence of surgical complications. However, complications, the most common of which were sinusitis and peri-implant soft tissue infection, may be underestimated due to the heterogeneity of the studies included.

## 1. Introduction

Zygomatic implants (ZIs), first introduced by Brånemark in 1988, were developed to rehabilitate patients presenting with severe maxillary atrophy or extensive defects secondary to trauma or pathological conditions, such as those following maxillectomy or tumor resection [1]. His original surgical technique (OST) was characterized by a large lateral osteotomy in the maxillary sinus with posterior crestal entry, typically in the second premolar region. This approach often resulted in a palatal emergence of the implant platform, with the implant trajectory following the zygomatic–alveolar crest through the sinus and terminating in stable anchorage within the zygoma [2]. Subsequently, Brånemark reported a 96% survival rate among a cohort of 52 zygomatic implants with a longitudinal follow-up exceeding five years, thereby establishing this technique as a feasible alternative to extensive and protracted pre-implant bone grafting procedures [3].

Over time, an evolution in the surgical methodology emerged, characterized by the utilization of extra-long titanium fixtures designed for bicortical engagement within the dense structure of the zygomatic bone, also focusing on the relationship with sinus anatomy [4,5,6].

Since 2006, Bedrossian and Chow et al. have conclusively validated that immediate functional loading of ZIs is clinically effective and reliable, providing substantial advantages compared to traditional grafting procedures [7,8].

The “sinus slot” technique, proposed by Stella and Warner, required a minimal antrostomy to guide implant drilling, avoiding a full sinus window and enabling placement in patients with pronounced buccal concavities approaching the maxillary sinus. [9].

In 2003, Boyes-Varley et al. further modified the original technique to improve surgical access and minimize postoperative complications. They also proposed modifications to implant head angulation and described ZIs as rescue solutions in cases of failure of tilted implants [10,11].

Subsequently, Malò et al. proposed an extra-maxillary approach, in which the implant trajectory was positioned entirely within the zygomatic bone and along the lateral wall of the maxillary sinus, thereby mitigating the risk of postoperative sinusitis [12]. Some clinicians have additionally performed crestal bone reduction as part of the surgical protocol, although this detail is inconsistently reported in clinical studies [13].

In 2010, Aparicio introduced the zygomatic anatomy-guided approach (ZAGA) classification, based on the radiographic analysis of 200 human jaws [14].

This approach has since been widely adopted in clinical practice and training, providing a systematic framework for adapting surgical techniques to various anatomical scenarios, ranging from flat maxillary walls to concave or severely atrophic jaws [15]. Although the implant survival rate has been widely documented by different authors, this outcome does not reflect the safety of using this type of rehabilitation [16]. In fact, each technical variation may carry a distinct risk profile; some are self-limiting, and others may require surgical revision, affecting patient quality of life. A structured evaluation of clinical complications is essential not only to inform surgical decision making but also to improve patient safety, optimize preoperative planning, and refine informed consent processes. Nonetheless, it remains essential to understand whether these different surgical protocols result in significantly different clinical outcomes, especially in terms of clinical complications, including paranasal sinus infections, intraoral soft tissue infections, nerve disorders, oroantral fistulas, extraoral hematomas, and prosthetic complications [17]. Furthermore, given the potential severity of certain adverse events—especially those involving the sinus or orbital region—zygomatic implant surgery carries relevant medico-legal implications. Inadequate case selection, insufficient radiological assessment, suboptimal surgical execution, or failure to adequately disclose specific procedural risks may expose clinicians to professional liability. Therefore, understanding whether specific surgical protocols are associated with different complication profiles is also crucial from a risk management and medico-legal perspective. The aim of this systematic review is to compare the clinical risk profiles associated with the different surgical techniques, such as OST and anatomically guided approaches for zygomatic implant placement, with particular emphasis on the incidence and nature of surgical, biological, and prosthetic complications.

## 2. Materials and Methods

### 2.1. Study Protocol

The present umbrella review was conducted and reported in accordance with the methodological guides of the Preferred Reporting Items for Systematic Reviews and Meta-analyses (PRISMA) 2020 checklist and the PRISMA checklist is provided in the Supplementary Materials [18].

The review protocol was registered on PROSPERO (CRD420251239499) [19].

The research question was formulated according to the PEO (population–exposure–outcome), a modified version of the PICO model [20,21].

The PEO question was articulated as follows:

P—Population: Patients treated with zygomatic implants;
E—Exposure: Insertion of zygomatic implants;
Outcomes: Surgical, biological, and prosthetic complications.

### 2.2. Search Strategy

**Figure 1:**
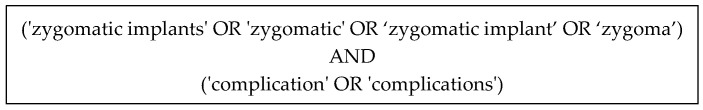
Keywords used for the search in the databases.

An electronic literature search was independently performed by two authors (F.D.A.) and (A.A.) across PROSPERO register, Scopus, MEDLINE/PubMed, BioMed Central, and the Cochrane Library. The search was conducted to identify systematic reviews addressing complications related to ZIs. Articles published in English up to 1 June 2025 were considered. The search strategy based on predefined keywords combined with Boolean operators was used as shown in Figure 1.

No grey literature was searched in this umbrella review.

In searching for the principal scientific databases, the filter ‘Review’ was applied within the Scopus repository, whereas the filters ‘Systematic Review’ and ‘Meta-analysis’ were employed in the MEDLINE/PubMed database. No filters were applied in the BioMed Central database, the Cochrane Library, or the PROSPERO register.

### 2.3. Study Selection and Eligibility Criteria

Data extraction was limited to studies published within the last ten years in order to focus exclusively on evidence related to current therapeutic approaches. The eligibility criteria for study selection were established as follows in Table 1 and Table 2. 

Duplicate records were removed using Zotero reference management software 8.0.4. The resulting titles were independently assessed by two reviewers (F.D.A. and A.A.), who screened the abstracts and selected the most pertinent studies for full-text evaluation. A third reviewer (F.G.) was consulted in cases of uncertainty or disagreement.

The reference lists of the reviews included were meticulously examined to identify any additional studies relevant to this umbrella review. We included only systematic reviews with and without meta-analysis that explicitly stated they were systematic reviews in the title and that followed a PRISMA chart.

No restrictions were imposed regarding the number or type of studies incorporated within the included systematic reviews.

### 2.4. Data Extraction and Collection

Data were obtained from two authors (F.D.A.) and (A.A.); however, a third reviewer (A.S.) was consulted in instances of uncertainty or interpretative ambiguity. Although formal inter-reviewer reliability metrics (e.g., kappa value) were not calculated prospectively, this process ensured rigor and minimized bias in study inclusion and data collection.

From all the articles, this information was extrapolated:

- Author, year of publication, reference, name of the journal, and study quality;
- Number and kind of included studies;
- Characteristics of the kind of surgical approach and complications;
- Main outcomes;
- Conclusions.

In detail, the outcomes analyzed in this review were implant osseointegration, implant success rate, implant survival, implant loss, and peri-implantitis.

### 2.5. Data Synthesis

Author, year of publication, reference, journal of publication, kind of review, study quality, number and design of included studies, outcomes, main results, and conclusions were organized in tabular format and synthesized through a narrative overview.

Data was synthesized using Microsoft Excel software 2019 (Microsoft Corporation, Redmond, WA, USA).

### 2.6. Assessment of Quality and Risk of Bias

The methodological quality of the systematic reviews included in this umbrella review was assessed using the AMSTAR tool (Ottawa Hospital Research Institute, Ottawa, Canada), accessible online: https://amstar.ca (accessed on 13 March 2026) [22].

## 3. Results

### 3.1. Study Selection

**Figure 2:**
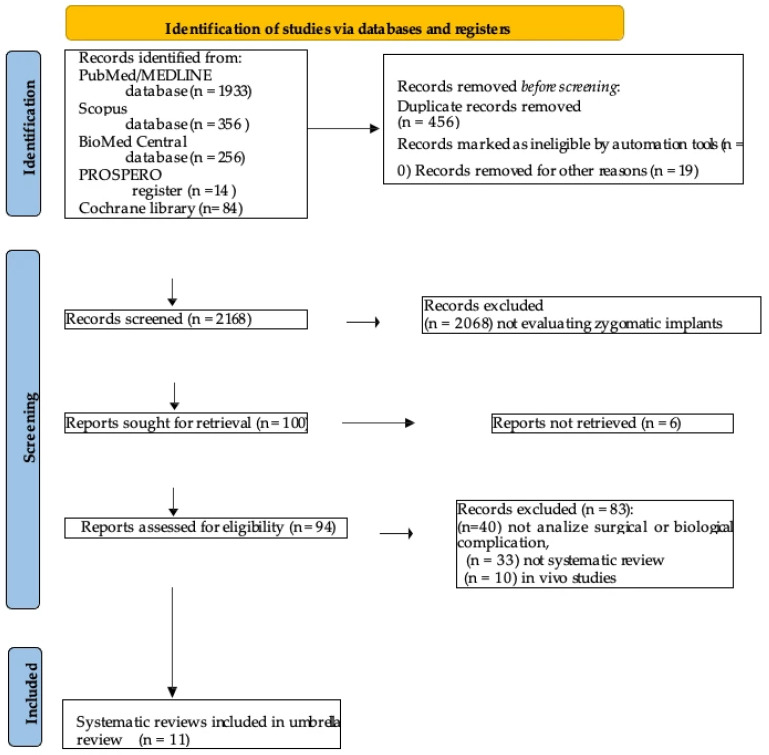
PRISMA 2020 flow diagram for new systematic reviews, which included searches of databases and registers only.

The PRISMA flowchart of the screening process is illustrated in Figure 2.

In the end, a total of 11 systematic reviews were included in this umbrella review.

### 3.2. Studies’ Characteristics and Qualitative Synthesis

Table 3 summarizes the characteristics of the included reviews All studies were published in English between 2016 and 2024.

Most of the complications were described after implant placement.

One of the most common complications was sinusitis, reported in all included systematic reviews.

### 3.3. The Main Outcomes Considered in This Umbrella Review

Table 4 summarizes the primary complications related to ZIs reported in the included studies, as well as several parameters analyzed by the authors (if available).

A meta-analysis was not conducted due to the substantial heterogeneity present across the datasets.

Various reviews incorporated into this umbrella synthesis evaluated the ZI complications using different surgical techniques. The most frequent biological complication was sinusitis.

### 3.4. Quality and Risk of Bias Assessment of Included Studies

Many studies were classified as low or moderate quality, and one was found to be of critically low quality, using the Assessing the Methodological Quality of Systematic Reviews (AMSTAR) 2 tool [22], as illustrated in Table 5.

**Table 5:** Level of evidence within included systematic reviews with meta-analyses according to the Assessing the Methodological Quality of Systematic Reviews (AMSTAR) 2 tool.

Level	Description	Kammerer [23]	Tavelli [16]	Chrcanovic [24]	Molinero-Mourelle [17]	Weber [25]	Lan [26]	Brennard Roper [27]	Gabriele [28]	Moraschini [29]	Tuminelli [30]	Gutiérrez Munoz [31]
High	No or one non-critical weakness										✓	
Moderate	More than one non-critical weakness		✓			✓		✓	✓	✓		
Low	One critical flaw with or without non-critical weaknesses	✓		✓	✓		✓					✓
Critically low	More than one critical flaw with or without non-critical weaknesses											

The level of evidence within the systematic reviews with meta-analyses included in this umbrella review is very important for the interpretation of the results, as reviews at high risk of bias show weaker evidence and conclusions than reviews at lower risk of bias.

## 4. Discussion

Zygomatic implants have profoundly transformed the management of severe jaw atrophy [32]. In this review, several studies compared the survival rates and complications profiles of ZIs placed using different techniques, particularly OST versus the ZAGA in patients with severe maxillary atrophy. Aparicio et al. analyzed OST and ZAGA approaches to evaluate the long-term performance of these surgical protocols and to assess the incidence of procedure-related complications. showing comparable clinical outcomes [33]. Chrcanovic et al. highlighted a 12-year cumulative survival rate of 95.21% based on 4556 ZIs, with most failures occurring during the early postoperative period [24]. The immediate loading rehabilitation turned out to be the primary advantage of this graft-free approach, enabling the patient’s oral function and aesthetic therapy following surgery, compared with conventional implant therapy. A marked difference in the distribution of delayed and immediate loading protocols was reported between the OST and the ZAGA, with rates of 77.7% versus 22.3% for OST and 10.4% versus 89.6% anatomy-guided [23]. Although this discrepancy likely reflects the predominance of more recent case series adopting anatomy-guided strategies, as well as advances in biomaterials, failure rates under immediate loading were 2.56% for OST and 1.75% for the anatomy-guided approach. These data were derived from the evaluation of 103 failures within a cohort of 4566 zygomatic implants, corresponding to an overall immediate-loading failure rate of 1.7% [24,34]. Despite the consistently high survival rates reported, operative, biomechanical, and prosthodontic complications require careful consideration.

### 4.1. Sinusitis

Among the complications observed after ZI surgery, sinusitis was found to be the most common adverse sequela. Maxillary sinus function can be assessed using the ORIS criteria, originally proposed by Aparicio et al., in combination with a clinical questionnaire [35]. Despite these assessments, it remains unclear whether zygomatic implants can trigger a foreign body reaction in the sinus membrane.

Etiologically, sinusitis may occur due to perforation of the Schneiderian membrane during the surgical procedure, movement of the ZI, blood ingress into the sinus cavity, or inadequate osseointegration at the coronal portion of the implant.

In a comparative study, the incidence of sinusitis was 27.2% in the group treated with the OST, in contrast to 3.7% observed in the anatomy-guided group. The comparative analysis by Aparicio et al. highlighted statistically significant differences between the two surgical approaches, with ZAGA associated with a reduced risk of maxillary sinus-related disease compared with the OST [36]. The incidence of sinusitis described suggests that surgical trajectory and sinus management play a pivotal role in reducing postoperative complications.

### 4.2. Local Soft Tissue Infections

One of the clinical signs of infection involving the superficial soft tissues surrounding zygomatic implants is peri-implant mucosa hyperplasia or recession, which may result in exposure of the implant surface or abutment [16]. At present, the concept of peri-implant disease in zygomatic implants remains poorly defined, as these implants achieve stabilization within the zygomatic bone tissue. Inadequate oral hygiene around the abutment was identified as the main etiological factor contributing peri-implant mucosal hyperplasia. For this reason, the design of the pontic in fixed prostheses—particularly the contour extending from the prosthetic base to the alveolar ridge crest—assumes critical significance [16,17]. To facilitate daily interdental hygiene procedures with dental floss, a designated "channel gap" may be incorporated within the prosthetic transition zone. The ORIS criteria indicate that ZI palatal emergence associated with an intrasinus approach can lead to bulky prostheses. If the abutment’s offset relative to the ridge exceeds 15 mm, the execution of daily hygiene measures may become considerably challenging [34,35]. Conversely, 52.9% (9 of 17) of patients included in the eligible studies exhibited recession at the implant–abutment interface when the ZAGA technique was utilized [11,28,37]. In Cawood–Howel groups V and VI or in ZAGA 4 and 5, the ZI placement may increase the risk of implant neck exposure on the vestibular aspect [26]. Another clinical manifestation that may occur is peri-implant recession, especially in cases of insufficient keratinized tissue, which may require the use of xenograft or autograft, as reported by Cikatricis et al. [38]. Nevertheless, the long-term effectiveness of soft-tissue regeneration around the ZI neck mandates further robust scientific evidence and underscores the need for standardized protocols for hygiene optimization.

### 4.3. Oroantral Fistula

The development of an oroantral fistula (OAF) in the context of ZIs is hypothesized to result from insufficient or compromised osseointegration between severely atrophic alveolar bone and the marginal region surrounding a palatally positioned ZI. This scenario can create patent communication between the maxillary sinus and the oral cavity, often leading to secondary sinusitis. OAF has been reported as a complication specifically associated with ZIs by Kammerer, Chrcanovic, and Tavelli, with incidence rates ranging from 1.5% to 7.5%. Consequently, strategic modification of the palatal design of the implant has been proposed as a potential measure to reduce the risk of fistula formation [16,23,24]. In the management of this condition, autologous platelet concentrates (APCs) are among the most extensively studied biomaterials [37,39]. Depending on the size of the communication, APCs may be applied either in monotherapy or in combination with resorbable membranes or connective tissue grafts. The final choice of therapeutic protocol also depends on patient compliance [40]. An oroantral communication secondary to fistula was observed in 31% of cases [23]. Furthermore, investigators suggested that excessive palatal placement of the ZI may predispose it to a localized deficiency in osseointegration within the marginal palatal bone. This deficiency is believed to compromise the load-bearing function of the implant and subsequently to induce transverse micromobility along the coronal segment of the ZI. One of the precautions taken to preserve residual alveolar bone in cases of ZI insertion is to avoid excessive countersinking or fracture of thin alveolar bone [23,24,41].

### 4.4. Paresthesia

Paresthesia represents a clinically significant complication following the insertion of ZIs. Postoperative paresthesia may arise from intraoperative overstretching of tissues during the surgical exposure of the zygomatic region.

Fifteen cases of paresthesia were documented in a systematic review by Kämmerer et al. [23]. Additionally, post-surgical edema can transiently induce a localized numbness in these innervated regions, a condition that typically resolves spontaneously within a short time frame.

Temporary infraorbital paresthesia is recognized as a common sequela of zygomatic implantation. This form of paresthesia is generally self-limiting, lasting from one week to three months post-surgery; nonetheless, certain instances may result in a permanent neurological deficit [26].

Davó et al. observed a notable variation, reporting that all 21 cases treated at a specific surgical facility experienced some degree of infraorbital paresthesia, while no instances were recorded at alternative locations [42,43]. One hypothesis supported by the authors to explain this high incidence was the use of wider mucoperiosteal flaps in the infraorbital region. Importantly, all patients in this cohort successfully recovered sensory perception without residual paresthesia within three months after surgery [23,43].

### 4.5. Facial Hematoma and Lip Laceration

The incidence of surgical complications has rarely been examined in depth across most clinical investigations, suggesting that the reported frequencies for both techniques may underestimate their actual prevalence.

The most reported postoperative sequela is facial hematoma, likely resulting from the extensive surgical exposure required within the zygomatic arch and adjacent regions. In addition, postoperative lip laceration has been observed; this complication is attributed to the limited oral opening often encountered and the use of the elongated drill instruments necessary for ZI placement, which can induce trauma to the lips if adequate soft tissue protection is not ensured. Clinicians must inform patients presenting with reduced dentition or restricted mouth opening that the osteotomy and drilling phase may present greater technical challenges compared with those encountered in fully edentulous individuals [23,38].

### 4.6. Ocular Complications

Orbital penetration is a complication reported by the authors in 30% of cases, resulting in permanent ocular motility deficit [38,44,45]. Drilling from the maxilla at a superolateral angle may breach the orbit during osteotomy in the inferolateral region [42]. However, oblique perforations through the superomedial region of the zygomatic bone are not reported to cause long-term ocular deficits [42,46,47]. More medial or deeper penetrations can damage the lateral or inferior rectus muscles and lead to intraorbital adhesions, reducing ocular motility [44]. Most penetrations were detected during or immediately after surgery [38,44,45], with only one case detected post-operatively on a follow-up CBCT scan [46]. Orbital penetration followed by orbital ZI placement resulted in damage to the lateral rectus and/or inferior oblique in 60% of those cases, causing loss of ocular motility, esotropia, or diplopia despite implant removal [45,48]. Some cases resulted in minimal consequences [46]. No globe ruptures have been reported, but this remains a theoretical risk given reported extraocular muscle injury. To reduce the risk of globe penetration, Topilow et al. recommended the placement of a protective plate in the lower conjunctival fornix [44]. In addition, adopting the extrasinus technique rather than the intrasinus technique may improve visualization of the drill during osteotomy, thereby lowering the risk of orbital penetration. One limitation of the intrasinus approach is poor visibility of the implant during its passage through the maxillary sinus [3], whereas the extrasinus technique allows greater control of the osteotomy due to improved visualization [28]. Given the high risk of long-term ocular deficits in cases of orbital penetration, Zielinski et al. proposed a classification of the zygomatic orbital floor (ZOF) to assist preoperative surgical planning and bone structure assessment [49]. These rare but severe complications may be reduced through the use of dynamic navigation systems (DNS) [28].

Following instrument calibration, the DNS provides real-time three-dimensional positioning of the drill, guiding its orientation within the bone. However, further studies are required to validate the effectiveness of this technology in reducing the risk of orbital perforation, a severe complication detectable on postoperative CBCT [50].

The precise incidence of orbital penetration during the surgical placement of ZI remains difficult to ascertain. Current methodological approaches likely underestimate this incidence, primarily because relevant clinical studies were not specifically designed for rigorous analysis of orbital penetration. This design limitation may consequently result in subtle but systematic under-reporting of cases classified as adverse surgical outcomes.

In cases of orbital penetration, prompt and tailored intervention is required [38,44]. Implant removal is the initial step, followed by management of ocular complications such as esotropia, strabismus, and drainage of hematomas. If esotropia persists after implant removal, strabismus surgery should be considered, as it improved but did not fully resolve symptoms in all three cases [44].

### 4.7. Prosthetic Complications

Prosthetic complications were reported more frequently in cases utilizing the OST compared to the ZAGA. These sequelae included a variety of mechanical and structural complications, including abutment screw loosening or fracture, framework or occlusal surface fracture, and various aesthetic deficiencies. Aparicio et al. reported that the occlusal surface fractures of acrylic and porcelain were the most frequent prosthetic complication [29]. The OST approach in fact showed that the implant emerged more palatally than natural dentition, and bulky restorations at the palatal aspect of the abutment connection were frequently observed. Some 4 of 13 OST studies reported this issue, which may hinder hygiene and cause speech discomfort relative to conventional restorations [25,26,27]. These data highlight the importance of using a ZAGA approach, based on prosthesis-guided implant placement, to minimize prosthetic complications.

### 4.8. Medico-Legal Aspects

Implant–prosthetic rehabilitation using zygomatic implants entails an intrinsic medical–legal risk. Owing to the highly invasive nature of the procedure and the surgical risks involved, clinicians are required to consider this option only in truly complex clinical scenarios. It is therefore essential to weigh up the overall risk–benefit ratio on a tailor-made case-by-case basis. After evaluating all possible therapeutic alternatives, such as various regenerative procedures or traditional removable prostheses, and after clearly outlining the potential complications associated with the procedure, the clinician should choose this technique only when no other viable options are available. This is crucial because complications arising from this treatment can be severe and, in some cases, may compromise any possibility of functional rehabilitation. Consequently, communication with the patient must be precise and comprehensive, detailing the potential intra- and postoperative risks, some of which can be highly debilitating. Furthermore, the operator’s surgical skill and experience should not be underestimated, as all the studies analyzed have shown this to be of fundamental importance in this procedure, a conditio sine qua non for its applicability.

### 4.9. Limitations of the Study and Distorted Quality of the Research

The systematic reviews included in this analysis consisted of studies that varied in design, sample size, follow-up duration, and lacked clear descriptions of surgical procedures. This heterogeneity prevented statistical comparisons and limited the possibility of performing subgroup analyses. Future studies supported by standardized protocols are needed to produce comparable data for clinical evaluation. Further studies regarding dynamic computer-assisted ZI surgery should include larger sample sizes and long-term outcomes to assess clinical effectiveness. Another important limitation is the quality and risk of bias assessment in the included systematic reviews. Several reviews included overlapping studies, such as Moraschini and Roper [27,29]. Overlapping studies included in systematic reviews represent a significant bias for this umbrella review, so the results should be analyzed and discussed with this significant bias in mind. Furthermore, the fact that many reviews were classified as being at high risk of bias should alert the reader to the fact that the data from this umbrella review are indicative, and further RCTs are needed to obtain systematic reviews with a low risk of bias. While independent screening by two reviewers and resolution of discrepancies by a third reviewer were performed, future updates of this review should incorporate these metrics to enhance transparency and reproducibility.

The search strategy is limited to the last 10 years because ZIs are primarily performed with the surgical techniques described in this umbrella review. Other obsolete techniques are no longer performed or are otherwise non-reproducible and could significantly bias this review.

In addition, only English-language articles were included because the most important international journals are in English, and we did not want to encounter translation issues. However, this could also be a limitation, as specified in the Limitations section.

Furthermore, no grey literature articles or registries other than PROSPERO were searched, which could also represent a potential source of bias in this umbrella review. The comparison between the original surgical technique and the anatomy-guided/ZAGA approaches appears clinically relevant. However, it should be noted that other confounding factors, such as follow-up period, surgeon experience, learning curve for performing the surgical procedure, and different patient populations, could partially influence this comparison.

## 5. Conclusions

The results of this umbrella review should be interpreted cautiously, given the marked heterogeneity in study design and the relatively small number of eligible studies or cohort studies addressing each specific topic. Nevertheless, current evidence indicates that ZI placement for the rehabilitation of severely atrophic edentulous maxillae is associated with a high implant survival rate and a low incidence of surgical complications. Among the adverse events, sinusitis and peri-implant soft tissue infection are the most prevalent. Complication rates for both techniques could be systematically underestimated, as most included studies did not clearly indicate whether specific complications were present. Both immediate and delayed loading protocols were described as yielding a high implant survival rate. Notwithstanding these methodological constraints, the available evidence suggests that zygomatic implants used for the rehabilitation of severely atrophic edentulous maxillae are associated with high survival rates and a limited frequency of surgical complications. Among the reported adverse events, sinusitis and peri-implant soft tissue infections appear to be the most prevalent. However, these complication rates should be considered carefully, as many of the included studies did not clearly state whether specific complications were systematically assessed or absent, potentially leading to under-reporting. Both immediate and delayed loading protocols were reported to achieve favorable implant survival outcomes.

The immediate loading protocol was more common when the ZAGA was employed compared to OST. A consistent finding across most of the analyzed studies highlighted the significant expertise and proficiency of the operating clinician as a key determinant of successful outcomes in these complex implant rehabilitation cases.

## Figures and Tables

**Table 1:** Inclusion criteria.

Inclusion Criteria
Only systematic reviews or meta-analyses
Review of the insertion of zygomatic implants and their surgical approach
Outcomes related to ZI complications (surgical, biological, and prosthetic)
Only English articles
Articles published in the last 10 years

**Table 2:** Exclusion criteria.

Exclusion Criteria
Non-systematic reviews with or without meta-analysis
Non-English articles
In vitro or animal reviews
Conference abstracts, letters, and commentaries

**Table 3:** Key characteristics of the included reviews: author, year, reference, journal, and type of review (with meta-analysis if applicable); number and design of included studies; surgical approach and reported complications; main outcomes and conclusions.

Author, Year, Reference Journal Meta-Analysis	Number and Design of Included Studies	Type of Surgical Approach and Complications	Outcomes	Conclusions
Kammerer, 2023 [23] Int J Implant Dentistry Systematic Review	24 studies (1 RCT and 6 PS; 17 RS;)	923 ZI (through OST) showed a survival of 90.3–100%, and 1302 ZI through AGA had a survival of 90.4–100% Sinusitis, 4.39% (more for OST than ZAGA) soft tissue infection, 4.35%; paresthesia, 0.55%. oroantral fistulas (13 cases for OST and 6 for ZAGA) 1.71%. and direct surgical complications, 1.60% Prosthetic complications were reported 56 and 104 cases in OST and ZAGA, respectively	Survival rate Local soft tissue infections Sinusitis Prosthetic complications Surgical complications Paresthesia Fistula	High implant survival rate and surgical complications within a minimum of 6 months are associated with ZI placement (through OST and ZAGA). Sinusitis and peri-implant soft tissue infection were the most common. The ZAGA method utilized a more immediate loading protocol than OST
Tavelli, 2022 [16] J Oral Implantol. Systematic Review	101 studied (69 RS, 29 PS, and 3 RCT)	No association was observed between survival rate and surgical technique, nor the surgical/restorative plan.	Survival rate Orbital cavity penetration Infection and oro-antral communication Labial laceration Hematoma Maxillary sinusitis Epistaxis	High implant survival rate associated with a low complication rate is observed in the case of ZI placement Several factors were found to be associated with the incidence of postoperative complications
Chrcanovic, 2016 [24] J Oral Maxillofac Surg Systematic Review	68 studies (1 RCT and 16 PS; 51 RS)	Sinusitis (2.4%) Soft tissue infection (2.0%) Oroantral fistulas (0.4%) Paresthesia (1.0%)	Survival rate Sinusitis Paresthesia Soft tissue infection Oroantral fistulas	The most common complication is sinusitis, even after several years from ZI placement
Molinero-Mourelle, 2016 [17] Med Oral Patol Oral Cir Bucal Systematic Review	14 studies (5 RS, 6 PS, 1 CS)	Sinusitis (3.9%) Failure in osseointegration (2.44%) Local infection (4%) Fistula at implant level (2%) Paresthesia (1.36%) Bruising (3.9%)	Surgical complications such as sinusitis and failure in osseointegration	This procedure depends on the clinician’s experience. The most common complications are sinusitis and failure in osseointegration of the zygomatic implant
Weber, 2024 [25] eCollection Systematic Review	24 studies ( 9 case reports. 1 case series. 9 cohort studies; 1 RCT; 4 SR)	Temporary infraorbital paresthesia (27 cases) Intraoperative orbital penetration (10 cases) Subconjunctival hemorrhage (5 cases)	Infraorbital paresthesia Intraoperative orbital penetration Orbital hematomas Extraocular muscle damage Subconjunctival hemorrhage Diplopia Orbital emphysema Infraorbital rim infections Periorbital fistulae	The most frequent complication is infraorbital paresthesia The most severe complication is orbital penetration, which is related to potentially even more severe sequelae This procedure should be performed by experienced clinicians
Lan, 2021 [26] Int J Oral Maxillofac Implants Systematic Review and Meta-analysis	11 studies (5 RS; 4 PS and 2 RCT)	Sinusitis (12%) Surgical-related complications (11 %) Local infection/injury (9%) Prosthetic complications (5%)	Sinusitis, Malposition and surgical guiding failure Implant survival rate	The procedure showed a high implant survival rate associated with complications that should not be underestimated
Brennard Roper, 2023 [27] Int J Implant Dent Systematic Review and Meta-analysis	18 studies (7 RS; 11 PS)	Sinusitis (14.2%) Implant failure (0.7%/year)	Long-term implant survival rate Postoperative complications Sinusitis	ZI could be a valuable therapy in case of the atrophic or resected maxilla, with a predictable survival rate. Sinusitis was the most frequent complication
Gabriele, 2023 [28] Int J Oral Maxillofac Implants Systematic Review and Meta-analysis	35 studies (1 RCT and 14 PS and RS)	Highest sinus-related and local infective complications with Brånemark technique (20%–34%) Dysesthesia/paresthesia/hematoma highest with sinus slot (10%) and extramaxillary (31%)	Postoperative complications in the different protocols and implant survival rate	The extrasinus approach presented the lowest percentage of both implant- and patient-level complications
Moraschini, 2023 [29] Clin Implant Dent Relat Res Systematic Review and Meta-analysis	RS and PS and RCT with at least 5 years of follow-up	Sinusitis was the most common complication	Implant survival rate Complication rate Sinusitis	The most frequent biological complication is sinusitis, which is most common with the IZI technique
Tuminelli, 2017 [30] Eur J Oral Implantol Systematic Review	CCS, PS, and RS of immediately loaded ZIs with a mean follow-up of 12 months.	Sinusitis Implant failure Perimplantitis	Implant survival rate Sinusitis	Immediately loaded ZI is a predictable therapy in cases of the maxilla affected by severe atrophy
Gutiérrez Munoz, 2021 [31] Biology (Basel) Systematic Review and Meta-analysis	46 studies (4 RCT and 19 PS; 20 RS; 3 CCS)	Sinus complications using the intrasinusal (7.2%) versus the extrasinusal technique (1.8%) Failure rate (0.69%) Prosthetic complications (4.9%)	Implant survival rate Prosthetic failure Peri-implantitis Sinusitis	ZI demonstrated a higher survival rate than conventional implants with a low incidence of prosthetic and biological complications

Abbreviations: zygomatic implant, “ZI”; randomized clinical trial, “RCT”; prospective studies “PS”; retrospective studies “RS”; case–control studies “CCS”; cohort studies “CS”; systematic review ‘SR’; original surgical technique (OST); zygomatic anatomy-guided approach (ZAGA).

**Table 4:** Characteristics of main outcomes and the main results from included studies.

Outcomes	Author, Years Reference	Main Features and Outcomes	Main Result
Surgical complications	Kammerer, 2023 [23]	The rates of paresthesia were 10.78% in the OST approach and 6.91% in the ZAGA approach, while the rates of direct surgical complications were 0.55% and 1.60%, respectively. Immediate loading had a prevalence of 22.3% in OST and 89.6% in the ZAGA.	ZI placement in case of edentulous maxillae affected by severe atrophy (both with OST and with AGA) is associated with a high implant survival rate and surgical complications (minimum 6 months follow-up). AGA utilized a more immediate loading protocol than OST.
Postoperative complications	Kammerer, 2023 [23]	Sinusitis, soft tissue infection and oro-antral fistulas resulted in the most common complications, at 9.53%, 7.50%, and 4.58%, respectively, in OST-treated patients. With AGA, the presenting complications were as follows: sinusitis, 4.39%; soft tissue infection, 4.35%; oroantral fistulas, 1.71%. The prevalence of the immediate loading protocol was 22.3% in OST and 89.6% in ZAGA.	ZI OST and AGA techniques in cases of severe maxillary atrophy showed a high implant survival rate and surgical complications within a minimum of 6 months of follow-up. Immediate loading was observed to be used more frequently in ZAGA.
Postoperative complications	Tavelli, 2022 [16]	Maxillary sinusitis, infection and oro-antral communication.	ZI therapy is a valuable option in case of maxilla affected by severe atrophy. The results highlighted a high implant survival rate associated with a low complication rate.
Prevalence of complications Survival rate	Chrcanovic, 2016 [24]	Sinusitis occurred in 2.4%; soft tissue infection occurred in 2.0%. Paresthesia of infraorbital and/or zygomaticofacial nerves after a ZI placement occurred in 1.0%. Oroantral fistulas occurred in 0.4%. There was a CSR of 95.21% over a 12-year period.	The most ZI failures or failures at the abutment connection occurred within 6 months of surgery. The most frequent complication was sinusitis, also several years after ZI placement.
Surgical complications	Molinero-Mourelle, 2016 [17]	Sinusitis (3.9%); Failure in osseointegration (2.44%) may increase the risk of oroantral communication; Local infection (4%); Fistula at implant level (2%); Paresthesia (1.36%) can vary linked to the surgeon’s expertise; Bruising (3.9%).	This procedure is strictly dependent on the clinician’s experience. The most common complications are sinusitis and failure of ZI osseointegration.
Ophthalmological complications	Weber, 2024 [25]	Temporary infraorbital paresthesia (27 cases); Intraoperative orbital penetration (10 cases); Orbital implant placement in 50% of cases; Subconjunctival hemorrhage (5 cases).	The most common complication is infraorbital paresthesia. The most severe complication is orbital penetration. This procedure should be performed by experienced surgeons.
Postoperative complications Implant survival rate	Lan, 2021 [26]	Sinusitis (12%); Malposition and surgical guiding failure (11 %); Local infection/injury (9%); Prosthetic complications (5%). Postoperative hematomas are common, with a mean spontaneous reabsorption time of 15 days.	The procedure showed a high implant survival rate.
Long-term implant survival rate Postoperative complications	Brennard-Roper, 2023 [27]	The prevalence of sinusitis ranged from 2.8% at 60 months mean follow-up to 36.4% at 141.6 months of mean follow-up. Implant failure was 0.7%/year. There was an implant survival rate of 96.2% at 6 years follow-up.	ZIs are a valuable treatment in cases of severe maxillary atrophy, with comparable survival rates to conventional implants. Sinusitis was the most reported factor related to implant loss.
Postoperative complications in the different described surgical techniques for implant placement Implant survival rate	Gabriele, 2023 [28]	The sinus slot technique resulted in the highest percentage of implant-level complications (16%). The most sinus-related and local infective complications occurred with the Brånemark technique (20% and 34%, respectively), while the extrasinus technique registered 0% in both categories. Dysesthesia/paresthesia/hematoma were the most common with sinus slot (10%) and extramaxillary (31%) but had a 0% rate with the extrasinus technique. There was no difference in survival rate between the four groups.	The extrasinus approach presented the lowest percentage of both implant- and patient-level complications. Surgical protocol selection is fundamental in reducing postoperative complications.
Implant survival rate Complication rate	Moraschini, 2023 [29]	They described sinutis, local infection and oroantral communication.	The most frequent biological complication is sinusitis, which is most commonly to the IZI technique.
Implant survival rate Prosthesis survival rate Sinus complications	Tuminelli, 2017 [30]	They described implant and prosthesis survival rate and potential surgical complications in zygomatic rehabilitations.	Immediately loading zygomatic implants for the restoration of the severely atrophic maxilla presents a viable alternative for the treatment of the atrophic maxilla.
Implant survival rate Prosthetic and sinus complications	Gutiérrez Munoz, 2021 [31]	The authors showed the cumulative incidence of sinus complications using the intrasinusal (7.2%) versus extrasinusal technique (1.8%), as well as the implant failure rate (0.69%) and prosthetic complication rate (4.9%), regardless of prosthetic treatment.	Zygomatic implants have higher survival rates than conventional implants. The incidence of prosthetic complications and sinusitis is low.

Abbreviations: zygomatic implant, “ZI”; original surgical technique (OST); zygomatic anatomy-guided approach (ZAGA).

## Data Availability

All data generated or analysed during this study are included in this article.

## Supplementary Materials

The following supporting information can be downloaded at https://www.mdpi.com/article/10.3390/diagnostics16060901/s1, Table S1: PRISMA 2020 Checklist
